# Bridging the Gap: Barriers to Access Autism Support for Immigrant and Refugee Communities in Canada and the United States

**DOI:** 10.1177/10436596261441556

**Published:** 2026-04-27

**Authors:** Hasina Amanzai, Angelina Stafford, Betty Qiuxuan Wang, Sepali Guruge, Cristina Catallo, Stephanie Nishi, Nancy Walton, Andrea Borges, Mushgan Sediq, Pheba Joy

**Affiliations:** 1 Toronto Metropolitan University, Ontario, Canada

**Keywords:** autism spectrum disorder, emigrants and immigrants, health services accessibility, cultural competency, health care disparities

## Abstract

**Objective:**

Autism spectrum disorder (ASD) affects one in 50 children in Canada and one in 31 children in the United States. This scoping review aimed to identify barriers immigrant families encounter when accessing ASD services.

**Method:**

Guided by the Joanna Briggs Institute framework, databases including CINAHL, Nursing & Allied Health Premium, PsycINFO, and Google Scholar were searched for peer-reviewed studies published within the past 10 years. A total of 12 studies examining immigrant parents’ experiences accessing ASD services through health or education systems were included.

**Results:**

Immigrant families reported barriers across cultural, systemic, and structural domains. Several studies also identified facilitators, such as bilingual providers and community-based support networks, which improved service access and trust.

**Conclusion:**

Findings indicate that immigrant families experience multiple, overlapping barriers that delay diagnosis and intervention. Addressing culturally responsive care, interpreter access, and service coordination is essential to improving equitable access to ASD services in Canada and the United States.

## Introduction

Autism spectrum disorder (ASD) is a neurodevelopmental condition characterized by persistent challenges in communication, social interaction, and restricted or repetitive behaviors across diverse racial, ethnic, linguistic, and socioeconomic groups (American Psychiatric Association, 2013, as cited in Imanpour, 2024). In Canada, ASD affects approximately one in 50 children (Public Health Agency of Canada, 2025a). In comparison, prevalence in the United States is higher, at approximately one in 31 children, based on recent national surveillance data (Centers for Disease Control and Prevention, 2025). Recent U.S. data indicate shifting prevalence patterns, with rates among Black, Hispanic, and Asian/Pacific Islander children now exceeding those of White children (Gallin et al., 2025). As prevalence continues to increase, ensuring equitable access to diagnosis and intervention has become an important public health priority.

Early identification and intervention during critical periods of brain plasticity are essential for improving developmental outcomes (Imanpour, 2024). Programs such as Early Intensive Behavioral Intervention (EIBI) can significantly improve developmental trajectories for children with ASD (Rivard et al., 2020). Although ASD can often be reliably diagnosed by 2 years of age, the median age of diagnosis remains closer to 4 years (Martinez et al., 2018). Delays in diagnosis and treatment can limit access to early intervention and negatively affect long-term outcomes (Fong et al., 2022).

Families frequently encounter barriers when accessing ASD services, including long wait times, fragmented service systems, and financial constraints. These challenges are often intensified for immigrant families, who may face additional barriers related to language differences, cultural stigma, unfamiliar health care systems, and limited social support networks (Khawar, 2024; Kim et al., 2024; Zhang, 2024). Children from racialized and minority ethnic groups are often diagnosed later and present with more severe symptoms than White peers (Sakai et al., 2019). In Canada, where immigrants comprise approximately 23% of the population, language barriers, cultural perceptions of ASD, and system navigation challenges may further delay service access (Lim, O’Reilly, Sigafoos, et al., 2020; Shanmugarajah et al., 2022; Statistics Canada, 2026). Despite growing recognition of these challenges, few reviews examine ASD service access among immigrant families. This scoping review maps individual, systemic, and structural barriers using an intersectionality-informed framework (Barbo, 2024; Casale et al., 2023).

## Method

### Ethical Consideration

Ethical approval was not required for this scoping review, as this review constitutes non-human subject research.

### Framework

This scoping review used the Population-Concept-Context (PCC) framework recommended by the Joanna Briggs Institute (JBI) to guide the research question and search strategy (Peters et al., 2020). The population included immigrant parents or caregivers of children with ASD who may face language, cultural, and systemic barriers to services (St. Amant et al., 2018). The concept examined barriers to accessing ASD services, including communication challenges and culturally incongruent care (Lim, O’Reilly, Londono, & Russell-George, 2020). The context included health care and educational service systems in Canada and the United States.

### Search Strategy

A scoping search was conducted across four electronic databases: CINAHL, Nursing & Allied Health Premium (NAHP), PsycINFO, and Google Scholar to identify literature examining barriers immigrant families face when accessing ASD services. No filters were applied to CINAHL, NAHP, or PsycINFO. In Google Scholar, a publication date filter (January 2014 to September 2024) was applied to prioritize recent evidence. Search terms were adapted to each database and included combinations of keywords related to ASD, immigrant populations, and health service accessibility. The full search strategies for each database are provided in Supplemental Appendix A (Table 1, Table 2). The search yielded 341 records: 81 from CINAHL, 178 from NAHP, 70 from Google Scholar, and 11 from PsycINFO. An updated search conducted on February 9, 2026, identified no additional eligible studies.

**Table 1.** Search Strategy of Each Database.

Database	Search strategy	Results
CINAHL	(“Asperger Syndrome” OR “Child Development Disorders, Pervasive” OR “Autism Spectrum Disorder”) AND (“Immigrants” OR “Parents”) AND (“Health Services Accessibility” OR “Communication Barriers”)	81
Nursing & Allied Health	“Autism spectrum disorder” AND “immigrants” AND “health services accessibility” AND “barriers”	178
Google Scholar Search 1	Immigrants; autism spectrum disorder; health services accessibility; barriers	60
Google Scholar Search 2	Immigrants; autism spectrum disorder; health services accessibility; communication barriers	10
PsycINFO	“Autism spectrum disorders” AND “barriers” AND “immigrants”	11
	Total	341

**Table 2.** Main Themes Breakdown.

Subfactors	Citations	Frequency
Individual factors		
Language barrier	Casale et al., 2023; Fong et al., 2022; Ijalba, 2016; Imanpour, 2024; Jaimes, 2022; Khanlou et al., 2017; Kim et al., 2024; Lim, O’Reilly, Londono, & Russell-George, 2020; Rivard et al., 2019; Sakai et al., 2019; Shanmugarajah et al., 2022	11
Cultural beliefs, difference, and stigma	Ijalba, 2016; Imanpour, 2024; Jaimes, 2022; Khanlou et al., 2017; Rivard et al., 2019, 2020; Shanmugarajah et al., 2022	7
Limited knowledge of ASD	Ijalba, 2016; Imanpour, 2024; Jaimes, 2022; Khanlou et al., 2017; Rivard et al., 2019, 2020; Shanmugarajah et al., 2022	7
Lack of social support and social isolation	Casale et al., 2023; Imanpour, 2024; Khanlou et al., 2017; Rivard et al., 2019, 2020	5
Emotional and mental strain	Casale et al., 2023; Fong et al., 2022; Kim et al., 2024; Lim, O’Reilly, Londono, & Russell-George, 2020	4
Limited health literacy	Lim, O’Reilly, Londono, & Russell-George, 2020	1
Systemic factors		
Anti-bilingualism and lack of culturally competent services	Casale et al., 2023; Fong et al., 2022; Ijalba, 2016; Imanpour, 2024; Khanlou et al., 2017; Kim et al., 2024; Rivard et al., 2019, 2020; Sakai et al., 2019; Shanmugarajah et al., 2022	10
Access to trained professionals/provider inexperience	Casale et al., 2023; Fong et al., 2022; Jaimes, 2022; Rivard et al., 2019, 2020; Sakai et al., 2019	6
Ineffective school policies	Fong et al., 2022; Rivard et al., 2020	2
Immigration status	Ijalba, 2016	1
Structural factors		
Financial constraints	Casale et al., 2023; Ijalba, 2016; Imanpour, 2024; Jaimes, 2022; Khanlou et al., 2017; Kim et al., 2024; Lim, O’Reilly, Londono, & Russell-George, 2020; Rivard et al., 2019, 2020; Sakai et al., 2019; Shanmugarajah et al., 2022	11
Fragmented systems and systemic complexity	Casale et al., 2023; Imanpour, 2024; Jaimes, 2022; Khanlou et al., 2017; Kim et al., 2024; Lim, O’Reilly, Londono, & Russell-George, 2020; Rivard et al., 2019	7
Long wait times	Casale et al., 2023; Fong et al., 2022; Imanpour, 2024; Khanlou et al., 2017; Lim, O’Reilly, Londono, & Russell-George, 2020; Rivard et al., 2019; Sakai et al., 2019	7
Transportation and geographic barriers	Casale et al., 2023; Fong et al., 2022; Imanpour, 2024; Jaimes, 2022; Khanlou et al., 2017; Rivard et al., 2020; Shanmugarajah et al., 2022	7
Limited service intensity	Rivard et al., 2020	1

### Inclusion Criteria

Studies were eligible if they examined immigrant parents or caregivers of children with ASD and explored barriers to accessing ASD-related services. Eligibility criteria were defined using the PCC framework recommended by the JBI. The population included immigrant families seeking ASD services for their children. The context included health care and educational service settings in North America, primarily Canada and the United States. Studies published in English between 2014 and 2024 were included to reflect contemporary service contexts. Qualitative, quantitative, mixed-methods, and systematic review designs were eligible. Only English-language studies were included to maintain consistency in data interpretation and due to limitations in reviewing non-English sources. Peer-reviewed articles were prioritized; however, relevant dissertations identified through Google Scholar and NAHP were also included as gray literature when they met the eligibility criteria.

### Exclusion Criteria

Studies were excluded if they did not focus on ASD, did not examine immigrant populations, or were conducted outside North America. Studies published prior to 2014 were also excluded. Additional exclusions included single-case studies with dual diagnoses, unclear immigrant status, or articles that did not align with the objectives of this review.

### Selection Process

**Figure 1.**
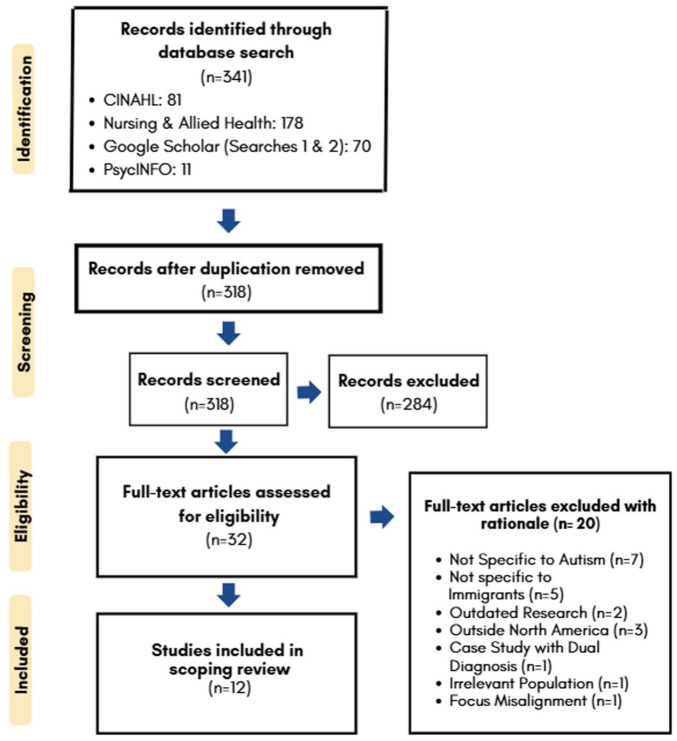
PRISMA Flow Diagram.

The review adhered to Preferred Reporting Items for Systematic reviews and Meta-Analyses extension for Scoping Reviews (PRISMA-ScR) guidelines. References were imported into Zotero for citation management (Corporation for Digital Scholarship, n.d.) and duplicate removal before being transferred to Covidence systematic review software (Veritas Health Innovation, n.d.) for screening. Two reviewers (AS and BW) independently screened titles and abstracts against the predefined inclusion criteria. Articles meeting the initial criteria were then assessed through full-text review by the same reviewers. Disagreements were resolved through discussion with a third reviewer (AB). Screening decisions were documented within Covidence to maintain an audit trail of reviewer decisions. A PRISMA-ScR flow diagram summarizing the identification, screening, and inclusion process is provided in Supplemental Appendix B (Figure 1).

### Data Extraction

For this scoping review, a data extraction tool was developed and managed using Google Sheets to systematically organize study characteristics and key findings. Data extraction was conducted by one reviewer and cross-checked by two additional reviewers to ensure consistency and accuracy. Discrepancies were discussed and resolved through consensus. The extraction tool captured study characteristics including study title, authors, year, country, setting, study aim, study design, population and sample characteristics, data collection and analysis methods, key results, study conclusions, and study limitations. These data were extracted consistently across all studies to support comparison and synthesis.

### Data Analysis

Included studies were reviewed to identify recurring patterns related to barriers immigrant families faced when accessing ASD services. An intersectionality framework was applied to explore how overlapping social factors, such as culture, language, and socioeconomic status, shaped these experiences. Extracted data were reviewed to identify common challenges across studies, which were grouped into subfactors representing recurring themes (e.g., language barriers, stigma, service access challenges). These were subsequently organized into three overarching domains: individual, systemic, and structural barriers. This process was conducted collaboratively by three reviewers (AS, BW, and AB), who discussed and refined theme groupings to ensure alignment with the study objectives. Data were synthesized using an iterative descriptive content analysis consistent with JBI scoping review methodology.

## Results

**Figure 2.**
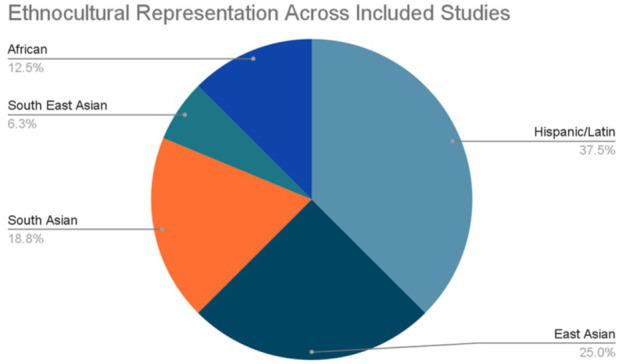
Ethnocultural Representation Across Included Studies.

The initial database search yielded 341 records, of which 23 were duplicates. After duplicate removal, 318 records underwent title and abstract screening. Of these, 32 articles were assessed through full-text review. Twenty studies were excluded for the following reasons: not specific to ASD (*n* = 7), not focused on immigrant populations (*n* = 5), publication date outside the eligibility window (*n* = 2), conducted outside North America (*n* = 3), case study with dual diagnosis not representative of the broader ASD population (*n* = 1), immigrant status not confirmed (*n* = 1), and misalignment with the review focus on ASD service access (*n* = 1). A total of 12 studies met the inclusion criteria. Participants represented diverse immigrant ethnocultural backgrounds, including Korean, Chinese, South Asian (including Tamil), Middle Eastern, East African, Hispanic/Latino, and Eastern European families. Languages represented across studies included Spanish, Farsi, Mandarin, Dari, Nepali, and Arabic. A summary of the included studies and ethnocultural representation is provided in Supplemental Appendix C (Figure 2).

The findings revealed barriers across three domains: individual/family, systemic, and structural factors. The most frequently reported barriers were language barriers (*n* = 11) and financial constraints (*n* = 11). Other commonly identified challenges included anti-bilingualism and lack of culturally competent services (*n* = 10), fragmented or complex service systems (*n* = 7), long wait times (*n* = 7), and transportation or geographic barriers (*n* = 7). Cultural stigma and differing interpretations of ASD (*n* = 7), limited knowledge of ASD (*n* = 7), and provider inexperience or limited access to trained professionals (*n* = 6) were also frequently reported. Social factors such as limited social support and social isolation (*n* = 5) and emotional or mental strain (*n* = 4) appeared with moderate frequency. Less commonly reported barriers included ineffective school policies (*n* = 2), limited health literacy (*n* = 1), immigration status concerns (*n* = 1), and insufficient service intensity (*n* = 1). A detailed breakdown of themes, subfactors, and frequency counts is provided in Supplemental Appendix D.

Most included studies used qualitative approaches (*n* = 9), while two used mixed-methods designs and one used a mixed experimental design. Language barriers, financial constraints, fragmented service systems, anti-bilingualism, and lack of culturally competent services were reported across nearly all ethnocultural groups. Cultural stigma and differing interpretations of ASD were particularly noted among South Asian Tamil families (Rivard et al., 2019; Shanmugarajah et al., 2022), Middle Eastern families (Casale et al., 2023; Imanpour, 2024), and East African parents (Khanlou et al., 2017). Limited ASD knowledge was more frequently reported among Hispanic mothers (Ijalba, 2016) and newcomer Asian families (Rivard et al., 2019; Sakai et al., 2019). Emotional and mental strain was especially noted among Korean and Middle Eastern caregivers (Casale et al., 2023; Fong et al., 2022; Kim et al., 2024), while social isolation was commonly reported among recent newcomers lacking extended family networks (Imanpour, 2024; Khanlou et al., 2017; Rivard et al., 2019). Transportation and geographic barriers disproportionately affected low-income families and those living in rural or poorly connected areas (Fong et al., 2022; Jaimes, 2022; Rivard et al., 2020).

### Individual and Family Factors

Cultural beliefs and stigma were commonly reported barriers influencing how immigrant families interpreted ASD. In several cultural contexts, ASD was viewed through moral or spiritual frameworks, such as divine punishment, karmic consequences, or threats to family reputation, contributing to shame and delayed help-seeking (Ijalba, 2016; Jaimes, 2022; Khanlou et al., 2017; Kim et al., 2024; Sakai et al., 2019; Shanmugarajah et al., 2022). Language barriers frequently limited effective communication with providers, contributing to misdiagnoses, delayed assessments, and reduced trust in health care systems (Casale et al., 2023; Fong et al., 2022; Shanmugarajah et al., 2022). Caregivers also reported emotional strain, including stress, guilt, and isolation while navigating complex service systems (Casale et al., 2023; Kim et al., 2024; Lim, O’Reilly, Londono, & Russell-George, 2020). Limited social support and low health literacy further affected families’ ability to navigate health care systems and understand ASD-related information (Imanpour, 2024; Khanlou et al., 2017; Lim, O’Reilly, Londono, & Russell-George, 2020; Rivard et al., 2019).

### Systemic Factors

Systemic barriers included limited access to trained professionals, particularly those who were bilingual or culturally competent (Jaimes, 2022; Sakai et al., 2019). Provider inexperience and limited ASD training contributed to misdiagnoses and delays in service delivery (Casale et al., 2023; Rivard et al., 2019). Families who relocated often lacked social networks to help navigate complex service systems (Casale et al., 2023). A lack of culturally competent services and conflicting guidance regarding language use further complicated access to care (Ijalba, 2016; Shanmugarajah et al., 2022). Delayed diagnoses were also reported, with many children identified after the optimal window for early intervention (Ijalba, 2016). Ineffective school policies, including limited accommodations, created additional barriers to educational support (Rivard et al., 2019). Immigration status also affected access, particularly for undocumented families facing restrictions in services and financial assistance (Ijalba, 2016).

### Structural Factors

Structural barriers included fragmented service systems and bureaucratic complexity, with families reporting disorganized processes, excessive paperwork, and poor coordination among providers that delayed access to care (Casale et al., 2023; Khanlou et al., 2017). Financial constraints were also common, including high therapy costs, limited insurance coverage, and insufficient public funding that disproportionately affected low-income families (Kim et al., 2024; Rivard et al., 2020; Shanmugarajah et al., 2022). Transportation and geographic barriers, particularly in rural areas, further limited consistent access to services (Fong et al., 2022; Khanlou et al., 2017). Long waitlists for publicly funded diagnostic and intervention services also delayed timely support (Casale et al., 2023; Lim, O’Reilly, Londono, & Russell-George, 2020; Rivard et al., 2019). Some families reported insufficient service intensity, with therapy hours falling below recommended levels (Rivard et al., 2020). Relocation disrupted continuity of care for some families, requiring them to restart diagnostic and intervention processes (Imanpour, 2024). Families also described difficulties navigating fragmented health care and educational systems without adequate guidance, as well as geographic and housing challenges that complicated access to appropriate services (Lim, O’Reilly, Londono, & Russell-George, 2020; Shanmugarajah et al., 2022).

## Discussion

This review examined how immigrant families experience barriers when accessing ASD-related services. Guided by an intersectionality-informed social determinants of health framework, we considered how individual, service-level, and broader system factors shape access to care. Although experiences varied across cultural and geographic contexts, several cross-cultural patterns emerged, including stigma, limited ASD knowledge, and challenges navigating service systems. These findings informed the policy and practice recommendations presented below.

### Individual and Family Factors

This review identified several individual and family-level barriers that shaped immigrant families’ experiences accessing ASD services. Language barriers were consistently linked to diagnostic delays, miscommunication with providers, and difficulties navigating service systems (Casale et al., 2023; Fong et al., 2022; Ijalba, 2016; Jaimes, 2022; Khanlou et al., 2017; Kim et al., 2024; Rivard et al., 2019; Shanmugarajah et al., 2022). Even when interpreters were available, limited ASD-specific knowledge sometimes resulted in incomplete communication (Imanpour, 2024; Shanmugarajah et al., 2022).

Cultural beliefs and stigma also shaped help-seeking. In several studies, ASD was interpreted through spiritual or moral frameworks, contributing to secrecy and delayed diagnosis (Fong et al., 2022; Imanpour, 2024; Jaimes, 2022; Kim et al., 2024; Sakai et al., 2019; Shanmugarajah et al., 2022). Concerns about family reputation and misconceptions about ASD further discouraged early engagement with services. Limited knowledge of ASD also contributed to delayed identification and intervention. Some parents were unfamiliar with developmental milestones or interpreted symptoms as temporary or culturally normative (Jaimes, 2022; Rivard et al., 2019, 2020). In the absence of clear, culturally relevant information, families often relied on informal sources that reinforced misconceptions (Ijalba, 2016; Imanpour, 2024; Khanlou et al., 2017; Shanmugarajah et al., 2022).

Emotional strain and social isolation further affected families’ engagement with services. Caregivers reported stress, depression, and guilt related to caregiving demands, immigration stressors, and unfamiliar systems (Casale et al., 2023; Fong et al., 2022; Kim et al., 2024; Lim, O’Reilly, Londono, & Russell-George, 2020). Migration-related disruptions to extended support networks also left many families without practical or emotional support during diagnosis and treatment (Casale et al., 2023; Imanpour, 2024; Khanlou et al., 2017; Rivard et al., 2019, 2020).

Limited health literacy also contributed to confusion when navigating referral systems and understanding ASD-related processes (Lim, O’Reilly, Londono, & Russell-George, 2020). Several studies also noted that culturally shaped gender roles positioned mothers as primary caregivers and system navigators, increasing emotional strain and sometimes delaying help-seeking when developmental concerns carried stigma. Together, these barriers often intersected, contributing to delayed diagnosis and reduced access to early intervention.

### Systemic Factors

Systemic barriers within health care, education, and social services also shaped immigrant families’ access to ASD care. A recurring concern was the lack of culturally competent and linguistically appropriate services. Families reported that programs often did not accommodate their linguistic or cultural needs, contributing to miscommunication, delays, and disengagement from services (Casale et al., 2023; Fong et al., 2022; Imanpour, 2024; Kim et al., 2024; Rivard et al., 2019). In some cases, clinicians discouraged bilingualism at home, incorrectly suggesting that exposure to two languages would hinder development (Ijalba, 2016; Kim et al., 2024).

Provider inexperience was another barrier. Some professionals lacked ASD-specific training, particularly in culturally diverse settings, which contributed to miscommunication and parents feeling misunderstood or excluded (Casale et al., 2023; Jaimes, 2022; Rivard et al., 2019; Sakai et al., 2019; Shanmugarajah et al., 2022). Frequent staff turnover and inconsistent care also required families to repeatedly report their child’s history (Fong et al., 2022; Rivard et al., 2020).

School-based barriers were also identified. Ineffective policies and limited coordination between educational and health services contributed to fragmented care and disruptions to family routines (Fong et al., 2022; Rivard et al., 2020) with regional disparities further restricting access. Immigration status created additional challenges. Families with precarious or unauthorized status sometimes avoided services due to fear of legal consequences or confusion about eligibility (Ijalba, 2016). Overall, systemic barriers embedded within service structures and policies continue to shape inequitable access to ASD care.

### Structural Factors

Structural barriers such as cost, service fragmentation, long wait times, transportation challenges, and limited service intensity were frequently reported. Financial strain was one of the most prominent concerns. Although Canada has publicly funded health care, many ASD-related services such as assessments and therapy are not fully covered, requiring families to pay out-of-pocket (Casale et al., 2023; Jaimes, 2022). Several studies noted that immigrant families were more likely to depend on partially funded programs or remain on long waitlists for services such as Applied Behavior Analysis therapy (Khanlou et al., 2017; Kim et al., 2024; Rivard et al., 2020; Sakai et al., 2019). Financial pressure was often compounded by housing instability and employment insecurity (Ijalba, 2016; Shanmugarajah et al., 2022). In response to delays, some families pursued private services despite unsustainable costs (Fong et al., 2022; Lim, O’Reilly, Londono, & Russell-George, 2020; Rivard et al., 2019).

Fragmented services and poor coordination were persistent concerns. Families often had to navigate health care, school, and social services independently (Casale et al., 2023; Imanpour, 2024; Jaimes, 2022; Khanlou et al., 2017). Complex paperwork and unclear processes made system navigation difficult (Rivard et al., 2019). Relocation between provinces or cities sometimes required families to restart assessments or lose previously established supports (Imanpour, 2024; Kim et al., 2024).

Long wait times further delayed access to care. Some families waited up to 2 years for publicly funded assessments (Casale et al., 2023; Fong et al., 2022; Imanpour, 2024), increasing the risk of missed opportunities for early intervention (Khanlou et al., 2017; Lim, O’Reilly, Londono, & Russell-George, 2020). Geography and transportation also limited access. Families without reliable transportation or living in poorly connected areas often had difficulty attending appointments (Casale et al., 2023; Jaimes, 2022). Multiple service locations added logistical strain, particularly for working caregivers or those facing language barriers (Fong et al., 2022; Imanpour, 2024; Rivard et al., 2020). Housing affordability further shaped access, as lower-cost areas were often located farther from services (Khanlou et al., 2017; Shanmugarajah et al., 2022). Service intensity presented another challenge. Programs such as EIBI sometimes offered fewer hours than recommended, limiting potential benefit (Rivard et al., 2020). Across studies, these structural constraints contributed to delays in diagnosis and disruptions in continuity of care.

### Implications for Policy, Practice, and Future Research

Building on the barriers identified in this review, several strategies may improve equitable access to ASD services for immigrant and refugee families. Workforce strategies should prioritize bilingual professionals, ASD-trained interpreters, and culturally responsive training to address communication barriers. Policy efforts should strengthen system navigation support, improve service integration, and expand funding to reduce fragmentation, long wait times, and financial strain. National standards for culturally responsive diagnostics and early intervention may help reduce regional inconsistencies and better reflect linguistic and cultural diversity. Collaboration across health care, education, settlement services, and community organizations may also improve coordination and continuity of care. Future research should evaluate culturally adapted interventions, improve data collection disaggregated by immigration status and ethnicity, and partner with immigrant-led organizations while considering gender, socioeconomic status, and migration experiences.

Clinicians, service providers, and educators can support improved access by adopting culturally responsive, family-centered approaches. Expanding access to bilingual professionals and ASD-informed interpreters may reduce communication barriers and better support caregivers navigating the health care system (Casale et al., 2023; Fong et al., 2022; Kim et al., 2024). Partnerships with cultural, faith-based, and immigrant-led organizations may also strengthen trust and engagement by connecting families with services that reflect their cultural background and preferred language.

Policymakers should prioritize system navigation supports, service integration, and funding reforms, including coverage for transportation and interpretation, and addressing gaps in intensive services such as Applied Behavior Analysis therapy (Khanlou et al., 2017; Kim et al., 2024; Rivard et al., 2019; Sakai et al., 2019). Future research should also focus on underrepresented groups, particularly refugees and racialized families, using longitudinal and participatory approaches to better understand how barriers evolve over time and to inform practical solutions. Evaluating culturally adapted interventions and system-level reforms will be important for improving access to ASD services (Fong et al., 2022; Imanpour, 2024; Shanmugarajah et al., 2022). Although one included study referenced both immigrant and refugee caregivers (Casale et al., 2023), refugee-specific barriers were not examined in depth, highlighting an important gap in the literature. These recommendations align with broader policy directions, including Canada’s emerging Autism Strategy, the Canadian Multiculturalism Act, and the World Health Organization (WHO) Framework on Integrated People-Centred Health Services (Government of Canada, 2025; Public Health Agency of Canada, 2025b; World Health Organization [WHO], 2016). Strengthening coordinated and equity-focused policies may improve timely and consistent access to ASD services for immigrant and refugee families.

### Limitations

This scoping review has several limitations. First, the review excluded studies published in languages other than English, which may result in excluding relevant findings in other languages. The studies included in this review were conducted in Canada and the United States; therefore, the findings are not generalizable to low- to middle-income countries with immigrant populations. Refugee families were notably underrepresented, with only one study briefly including them (Casale et al., 2023) and none examining refugee-specific barriers in depth. Certain ethnocultural groups, including Filipino, European, and Latin American families, appeared only once, limiting broader conclusions. Several barriers including limited health literacy, service intensity, immigration status concerns, and school policy challenges were reported in few studies, indicating limited evidence in these areas. As a result, existing research disproportionately reflects the experiences of Asian, Hispanic/Latino, and Middle Eastern families, while other groups and underexamined barriers warrant further attention. Although some barriers were universal, challenges varied across cultural, linguistic, and settlement contexts, underscoring the need for subgroup-specific considerations in ASD service planning.

## Conclusion

This scoping review synthesized evidence on barriers that immigrant and refugee families in Canada and the United States face when accessing ASD services for their children. Across studies, barriers at the individual, service, and structural levels contributed to delayed access to care. These included language differences, cultural stigma, provider inexperience, fragmented systems, long wait times, and financial strain. These challenges can delay diagnosis and intervention, particularly for low-income, racialized, and newcomer families navigating complex systems with limited social support. Improving access will require coordinated action across policy, clinical practice, and community systems. Strengthening funding models, interpreter services, culturally responsive care, and system navigation supports may reduce barriers and improve continuity of care. Future research should examine underrepresented groups, including refugee families, and evaluate culturally adapted service models to address persistent disparities in ASD access.

## Supplemental Material

sj-docx-1-tcn-10.1177_10436596261441556 – Supplemental material for Bridging the Gap: Barriers to Access Autism Support for Immigrant and Refugee Communities in Canada and the United States Supplemental material, sj-docx-1-tcn-10.1177_10436596261441556 for Bridging the Gap: Barriers to Access Autism Support for Immigrant and Refugee Communities in Canada and the United States by Hasina Amanzai, Angelina Stafford, Betty Qiuxuan Wang, Sepali Guruge, Cristina Catallo, Stephanie Nishi, Nancy Walton, Andrea Borges, Mushgan Sediq and Pheba Joy in Journal of Transcultural Nursing
