# Supplementary material for: Bridging the Gap: Barriers to Access Autism Support for Immigrant and Refugee Communities in Canada and the United States
Source: J Transcult Nurs. 2026 Apr 27;37(5):789–97. doi: 10.1177/10436596261441556 (PMC13478404; doi:10.1177/10436596261441556)
Supplement: Supplementary file 1 [file sj-docx-1-tcn-10.1177_10436596261441556.docx]

**Appendix A**

Table 1: Search Terms

| **Database** | **Search Terms** | **Filters/Limits Applied** |
| --- | --- | --- |
| CINAHL | (“Asperger Syndrome” OR “Child Development Disorders, Pervasive” OR “Autism Spectrum Disorder”) AND (“Immigrants” OR “Parents”) AND (“Health Services Accessibility” OR “Communication Barriers”) | None |
| NAHP | autism spectrum disorder AND immigrants AND health services accessibility AND barriers | None |
| PsycINFO | autism spectrum disorders AND barriers AND immigrants | Peer-Reviewed |
| Google Scholar (Search 1) | immigrants AND autism spectrum disorder AND health services accessibility AND barriers | January 2014-2024 |
| Google Scholar (Search 2) | immigrants AND autism spectrum disorder AND health services accessibility AND barriers AND communication barriers | January 2014-2024 |

**Appendix B**

**Appendix C**

Table 2: Summary of Articles

| **Authors** | **Country** | **Year** | **Study Design** | **Participants** | **Ethnic Group** | **Data Collection & Analysis** | **Setting** |
| --- | --- | --- | --- | --- | --- | --- | --- |
| Casale, Lina Marie; Gentles, Stephen J; McLaughlin, Janet; Schneider, Margaret | Canada | 2023 | Qualitative study using in-depth interviews. | 12 immigrant families. | Primarily South Asian and Middle Eastern families. | Semi-structured interviews with parents, analyzed using thematic coding. Data triangulated with service provider insights. | Community-based services for immigrant families with children diagnosed with autism. |
| Fong, Vanessa C; Lee, Bo Sang; Iarocci, Grace | Canada | 2022 | Qualitative, community-engaged approach using semi-structured interviews. | 20 Korean immigrant parents of children with autism spectrum disorder (ASD). | Korean immigrant families. | Semi-structured interviews conducted in English or Korean.  Thematic analysis using NVivo software.  Constructivist grounded methodology. | Autism services in Korean immigrant families in British Columbia, conducted by researchers from Simon Fraser University. |
| Ijalba, Elizabeth | US | 2016 | Qualitative phenomenological interviews conducted with thematic analysis. | 22 Hispanic immigrant mothers who are primary caregivers for preschool-aged children with ASD. | Hispanic mothers from Mexico, Dominican Republic, Ecuador, and Peru. | Conducted three in-depth interviews with each participant (66 interviews total).  Themes were derived through a coding frame, inductive analysis, and narrative development.  Member checking and peer examination were employed to enhance reliability and validity. | Conducted in a preschool setting in New York City. Principal Investigator: Elizabeth Ijalba, Speech-Language Pathology discipline. |
| Imanpour, Sara | US | 2024 | Qualitative interpretive study using grounded theory methodology. | 16 immigrant mothers with LEP and preschool-aged children diagnosed with autism. | Diverse immigrant mothers speaking Spanish, Farsi, Mandarin, Dari, Nepali, and Arabic. | - Semi-structured interviews conducted in participants' native languages with translation support.  - Data coded using the constant comparative method and analyzed with the Kilbourne health disparity framework. | Health and early intervention services for families with Limited English Proficiency (LEP) in various states across the U.S.; Principal Investigator focuses on health disparities. |
| Jaimes, Alma | US | 2022 | Extended literature review with qualitative data integration. | No primary participants; review focused on Hispanic immigrant parents of children with ASD. | Hispanic and Latino immigrant families, including subgroups from Mexico, Central America, and the Caribbean. | Synthesizing existing literature and case studies.  Examination of systemic barriers, cultural influences, and educational frameworks. | Educational and psychological research with a focus on Hispanic immigrant families navigating autism-related challenges. |
| Khanlou, Nazilla; Haque, Nasim; Mustafa, Nida; Vazquez, Luz Maria; Mantini, Anne; Weiss, Jonathan | Canada | 2017 | Qualitative descriptive approach with in-depth interviews | 21 immigrant mothers with children diagnosed with ASD participated. | Participants represented regions including Asia (e.g., China, India, Philippines), Europe (e.g., Ukraine, Poland), and Latin America (e.g., Costa Rica, Trinidad and Tobago). | Semi-structured interviews (60–90 minutes) conducted via telephone.  Interviews transcribed and analyzed using thematic analysis aligned with House's four social support domains.  Triangulation and member checking employed to ensure rigor. | Research conducted in the Greater Toronto Area (GTA), examining barriers immigrant mothers face in accessing services for children with Autism Spectrum Disorder (ASD). Principal investigator associated with Women's Health Research Chair in Mental Health, York University |
| Kim, Hyeyoung; Kim, Sohyun An; Lee, Han; Dodds, Robin | US | 2023 | Qualitative; thematic analysis of semi-structured interview | 11 first-generation Korean immigrant mothers | Korean immigrant families | Semi-structured interviews conducted in Korean, translated into English.  Analysis conducted via thematic coding using Dedoose software, focusing on recurring patterns and themes. | Autism diagnosis and service access; Korean immigrant families |
| Lim, Nataly; O'Reilly, Mark F.; Londono, Fabiola Vargas; Russell-George, Adrienne | US | 2020 | Mixed design with a multiple baseline across families and replication using an alternating treatments design for one family. | Four families (parents and children with ASD). | Hispanic (Spanish-speaking immigrant parents). | Parents’ instructional fidelity and children’s independent dressing skills were measured across baseline, intervention, post-intervention, maintenance, and follow-up phases.  Data analyzed using task analysis scores, interobserver agreement, and social validity measures. | Home-based intervention in collaboration with Spanish-speaking immigrant parents; Principal Investigator affiliated with the University of Texas at Austin. |
| Rivard, Mélina; Millau, Marie; Magnan, Charlotte; Mello, Catherine; Boulé, Mélina | US | 2019 | Cross-sectional mixed-methods study, combining quantitative surveys and qualitative interviews. | 184 families of children diagnosed with ASD | Diverse sample, including Hispanic, African American, Asian, and Caucasian families. | - Quantitative: Surveys administered to families assessing service access, satisfaction, and perceived barriers.  - Qualitative: Semi-structured interviews analyzed using thematic coding. | Various healthcare and educational settings focused on autism services; Principal Investigator specializes in developmental disabilities and family support systems. |
| Rivard Mélina; Millau Marie; Mello, Catherine; Clément Céline; Mejia-Cardenas, Catalina; Boulé Mélina; Magnan, Charlotte | Canada | 2020 | Mixed-methods study using surveys and semi-structured interviews | 25 immigrant families of children diagnosed with ASD who had participated in EIBI programs. | Participants represented a variety of cultural and linguistic backgrounds, including South Asian, Middle Eastern, East Asian, and African families. | - Surveys were administered to collect quantitative data on satisfaction and barriers.  - Interviews were conducted to gather qualitative insights.  - Data were analyzed thematically and descriptively. | Community-based Early Intensive Behavioral Intervention (EIBI) programs in Ontario; research conducted within developmental psychology and educational settings. |
| Sakai, Christina; Mulé, Christina; LeClair, Amy; Chang, Frances; Sliwinski, Samantha K.; Yau, Yoyo; Freund, Karen M. | US | 2019 | Qualitative study using semi-structured interviews. | 16 parents and 16 providers. Parents had children diagnosed with autism; providers worked with Chinese immigrant families. | Chinese immigrant families; providers included 75% Asian (Chinese). | Interviews were conducted in Cantonese or Mandarin for parents and in English for providers.  Data analyzed using modified grounded theory.  Audiotaped interviews were transcribed, translated, and coded by bilingual researchers. | Large academic medical center in the Northeast region of the United States, with support from the Chinatown Neighborhood Center. Research led by experts in pediatrics, community health, and translational sciences. |
| Shanmugarajah, Kajaani; Rosenbaum, Peter; Di Rezze, Briano | Canada | 2022 | Descriptive qualitative study | 8 participants (Sri Lankan Tamil mothers of children diagnosed with ASD). | Sri Lankan Tamil | Semi-structured interviews analyzed using Graneheim and Lundman’s content analysis framework. | Conducted at the SAAAC Autism Centre in Scarborough, Ontario; focus on occupational therapy and cultural competence. |

**Appendix D**

Table 3: Main Themes Breakdown

| **Sub-Factors** | **Citations** | **Frequency** |
| --- | --- | --- |
| **Individual Factors** | | |
| Language Barrier | Casale et al., 2023; Fong et al., 2022; Ijalba, 2016; Imanpour, 2024; Jaimes, 2022; Khanlou et al., 2017; Kim et al., 2024; Lim et al., 2020b; Rivard et al., 2019; Sakai et al., 2019; Shanmugarajah et al., 2022 | 11 |
| Cultural Beliefs, Difference and Stigma | Ijalba, 2016; Imanpour, 2024; Jaimes, 2022; Khanlou et al., 2017; Rivard et al., 2019; Rivard et al., 2020; Shanmugarajah et al., 2022 | 7 |
| Limited Knowledge of ASD | Jaimes, 2022; Ijalba, 2016; Imanpour, 2024; Khanlou et al., 2017; Rivard et al., 2019; Rivard et al., 2020; Shanmugarajah et al., 2022 | 7 |
| Lack of Social Support and Social Isolation | Casale et al., 2023; Imanpour, 2024; Khanlou et al., 2017; Rivard et al., 2019; Rivard et al., 2020 | 5 |
| Emotional and Mental Strain | Casale et al., 2023; Fong et al., 2022; Kim et al., 2024; Lim et al., 2020b | 4 |
| Limited Health Literacy | Lim et al., 2020b | 1 |
| **Systemic Factors** | | |
| Anti-bilingualism & Lack of Culturally Competent Services | Casale et al., 2023; Fong et al., 2022; Ijalba, 2016; Imanpour, 2024; Khanlou et al., 2017; Kim et al., 2024; Rivard et al., 2019; Rivard et al., 2020; Sakai et al., 2019; Shanmugarajah et al., 2022 | 10 |
| Access to Trained Professionals/Provider Inexperience | Casale et al., 2023; Fong et al., 2022; Jaimes, 2022; Rivard et al., 2019; Rivard et al., 2020; Sakai et al., 2019 | 6 |
| Ineffective School Policies | Fong et al., 2022; Rivard et al., 2020 | 2 |
| Immigration Status | Ijalba, 2016 | 1 |
| **Structural Factors** | | |
| Financial Constraints | Casale et al., 2023; Ijalba, 2016; Imanpour, 2024; Jaimes, 2022; Khanlou et al., 2017; Kim et al., 2024; Lim et al., 2020b; Rivard et al., 2019; Rivard et al., 2020; Sakai et al., 2019; Shanmugarajah et al., 2022 | 11 |
| Fragmented Systems and Systemic Complexity | Casale et al., 2023; Imanpour, 2024; Jaimes, 2022; Khanlou et al., 2017; Kim et al., 2024; Lim et al., 2020b; Rivard et al., 2019; | 7 |
| Long Wait Times | Casale et al., 2023; Fong et al., 2022; Imanpour, 2024; Khanlou et al., 2017; Lim et al., 2020b; Rivard et al., 2019; Sakai et al., 2019 | 7 |
| Transportation and Geographic Barriers | Casale et al., 2023; Fong et al., 2022; Jaimes, 2022; Imanpour, 2024; Khanlou et al., 2017; Rivard et al., 2020; Shanmugarajah et al., 2022 | 7 |
| Limited Service Intensity | Rivard et al., 2020 | 1 |
